# Clinical characteristics and outcomes of ischemic stroke despite appropriate oral anticoagulation for atrial fibrillation: A systematic review and meta-analysis of real-world studies

**DOI:** 10.1007/s10072-025-08734-2

**Published:** 2025-12-22

**Authors:** Francesca Gabriele, Federico De Santis, Maria Grazia Vittorini, Lucio D’Anna, Giovanni Merlino, Francesco Bax, Raffaele Ornello, Simona Sacco, Matteo Foschi

**Affiliations:** 1 Department of Biotechnological and Applied Clinical Sciences, University of L’Aquila, Via Vetoio, 67100 L’Aquila, Italy; 2 Department of Brain Sciences, Imperial College London, London, UK; 3 Department of Stroke and Neuroscience, Charing Cross Hospital, Imperial College, Healthcare NHS Trust, London, UK; 4 Clinical Neurology, Udine University Hospital and DAME, University of Udine, Udine, Italy; 5 Stroke Unit and Clinical Neurology, Udine University Hospital, Udine, Italy; 6 Department of Neurology, J Philip Kistler Research Center, Massachusetts General Hospital and Harvard Medical School, Boston, MA USA; 7 Department of Neuroscience, Neurology Unit - Stroke Unit, S.Maria delle Croci Hospital, AUSL Romagna, Ravenna, Italy

**Keywords:** Meta-analysis, Oral anticoagulation, Direct oral anticoagulant, Vitamin k antagonists, Characteristics, Outcomes, Prognosis

## Abstract

**Background:**

Despite appropriate oral anticoagulants (OACs) use to prevent cardioembolism in atrial fibrillation (AF) and thromboembolic conditions, some patients still experience ischemic stroke.

**Objectives:**

This systematic review and meta-analysis aimed to investigate clinical characteristics and outcomes of ischemic stroke occurring despite appropriate OACs use for atrial fibrillation, comparing direct oral anticoagulants (DOACs) with vitamin K antagonists (VKAs).

**Methods:**

A systematic search of PubMed and Scopus identified observational cohort studies published in English until October 2, 2024, on ischemic stroke patients on appropriate OAC doses. We utilized random-effects modeling to assess the impact of stroke severity, anticoagulant type, and revascularization on in-hospital mortality, 90-day functional recovery (modified Rankin Scale [mRS] 0–2), 90-day stroke recurrence, and intracranial hemorrhage (ICH).

**Results:**

Out of 25,985 articles screened, 11 studies (14 cohorts) involving 10,624 patients were included. The mean age was 76 years, with median National Institute of Health Stroke Scale (NIHSS) score of 5.1. Prevalent risk factors included hypertension (78.3%), dyslipidemia (44.3%), and prior stroke or TIA (55.6%). No significant baseline differences were found by OAC type, except for higher coronary heart disease prevalence in the VKA group. DOAC patients showed better 90-day functional outcomes than VKA patients (60.4% vs. 35.7%; p = 0.048). Meta-regression did not identify significant predictors for in-hospital death or 90-day favorable outcomes.

**Conclusions:**

Ischemic stroke patients despite OACs exhibited a high burden of vascular risk factors and moderate stroke severity. DOAC-related strokes showed better 90-day functional outcomes than those on VKA. Further research on long-term prognosis is needed.

**Supplementary Information:**

The online version contains supplementary material available at 10.1007/s10072-025-08734-2.

## Introduction

Oral anticoagulation (OAC) is the cornerstone of stroke prevention in patients with atrial fibrillation (AF) or other thromboembolic conditions, as recommended by international guidelines [1, 2]. The selection of the appropriate anticoagulant and its dosage is guided by individual stroke risk, bleeding risk, renal function, and potential drug interactions to optimize stroke prevention while minimizing adverse effects. In recent years, direct oral anticoagulants (DOACs) have largely replaced vitamin K antagonists (VKAs) due to their more predictable pharmacokinetics, fewer dietary and drug interactions, and a lower risk of intracranial hemorrhage, while maintaining at least non-inferior, if not superior, efficacy in preventing thromboembolism. [3]

Despite adherence to guideline-directed therapeutic OAC therapy, a subset of patients (approximately 1–2% of those with AF) still experience an acute ischemic stroke each year [4]. This phenomenon raises concerns about the mechanisms underlying anticoagulant failure. While some cases may be attributed to poor adherence, temporary discontinuation, drug interactions, or competing stroke mechanisms such as small vessel disease or atherosclerosis,[5–7] others occur despite fully appropriate anticoagulation.

Several cohort studies have examined the short-term outcomes of ischemic strokes in patients on OACs, but these analyses often include heterogeneous populations with varying levels of anticoagulation adequacy, making it difficult to isolate the effects of fully appropriate anticoagulation [8–10]. Data from the Get With The Guidelines–Stroke (GWTG-Stroke) registry in the United States suggest that among AF patients experiencing an acute ischemic stroke, those who had received therapeutic anticoagulation prior to the event had lower stroke severity and reduced in-hospital mortality compared to those not on OACs [11]. Similarly, findings from the Korean Stroke Registry (KSR) indicate that appropriate pre-stroke anticoagulation is associated with milder stroke severity and improved functional outcomes at discharge compared to no anticoagulation or subtherapeutic treatment [12]. However, there is still a gap in understanding the clinical profile of ischemic strokes that occur despite fully appropriate OAC therapy. A thorough characterization of this population is essential to uncover residual stroke risk factors, improve prognostic assessment, and enhance anticoagulation strategies for high-risk patients.

Hence, we conducted a systematic review and meta-analysis to investigate the clinical features and outcomes of ischemic stroke occurring despite appropriate oral anticoagulation for guideline-recommended doses and indications. Furthermore, we analyzed the impact of direct oral anticoagulants (DOACs) versus vitamin K antagonists (VKAs).

## Material and methods

### Search strategy and selection criteria

The present systematic review and meta-analysis followed the Preferred Reporting Items for Systematic Reviews and Meta-Analysis (PRISMA)[13] guidelines and the Cochrane handbook for Systematic Reviews of Interventions [14]. The protocol was registered in PROSPERO with code CRD42024571231 on September 5, 2024. We selected articles and planned a search strategy according to the Population-Exposure-Outcome (PEO) chart reported in Table 1.

**Table 1:** Population-Exposure-Outcome chart

Population	Patients with acute ischemic stroke and atrial fibrillation
Exposure	Appropriate oral anticoagulation at the time of ischemic stroke onset
Outcome	In-hospital death due to any cause, 90-day death due to any cause, 90-day functional independence (mRS of 0 to 2), 90-day ischemic stroke recurrence, ICH

Abbrev. ICH: intracranial hemorrhage; mRS: modified Rankin Scale; NIHSS: National Institutes of Health Stroke Scale;

We made a literature search on articles published up to October 2, 2024 on PubMed (MEDLINE) and EMBASE. The search strings used for the search are reported in Supplementary Table 1. The literature search was performed independently by 8 authors (MF, RO, GM, FB, FG, FDS, LD, MGV) using Rayyan Systematic Review web-based tool [15]. Then, the same authors selected the articles after examining the full text. Disagreements on eligibility were resolved by consensus among all involved authors.

We included observational cohort studies that examined patients with acute ischemic stroke despite receiving an appropriate dose of oral anticoagulants for AF, as defined by drugs classified under the B01AF (direct oral anticoagulants) or B01AA (vitamin K antagonists) categories of the Anatomical Therapeutic Chemical (ATC) classification [16]. Specifically, for VKAs, anticoagulation was considered appropriate if the international normalized ratio (INR) at admission was ≥ 1.7 or ≥ 2.0, depending on the cut-off values used in the analyzed studies. The specific INR cut-off applied in each study is detailed in Supplementary Table 2. Although the accepted therapeutic INR for AF is typically ≥ 2.0 (or higher for patients with coexisting valvular defects or artificial valves)[17], we recognize that, for evaluating potential therapeutic approaches within this population, an INR cutoff of 1.7 is relevant. This threshold was chosen as it corresponds to the cut-off adopted in a large prospective study investigating the characteristics and outcomes of patients with ischemic stroke to define therapeutic anticoagulation with VKAs [18]. Additionally, evidence from RCTs and observational studies showed that a residual anticoagulant effect is still present up to an INR of 1.7 and this evidence forms the basis for guideline recommendations supporting intravenous thrombolysis as a safe option within this range [19–22]. For DOACs, appropriateness was assessed based on compliance with guideline-recommended on-label dosing [23]. In cases where studies did not report INR at admission for patients on VKAs or did not specify whether patients on DOACs were receiving appropriate on-label dosing, those patients were excluded from the analysis (wrong exposure). Other exclusion criteria included studies involving the wrong population (i.e., patients without ischemic stroke), the wrong study design (i.e., studies that did not report data on clinical characteristics or post-stroke outcomes), or the wrong article type (i.e., case reports, case series, letters, editorials, comments, narrative or systematic reviews, and meta-analyses). Studies published in languages other than English were also excluded. Furthermore, to improve effect size precision and enhance the overall reliability of the meta-analysis findings, we excluded studies or populations with limited data (< 50 patients).

### Quality assessment

The quality of cohort studies included in the meta-analysis was assessed with the Newcastle–Ottawa scale scoring instrument [24]. According to this tool, we evaluated three aspects: selection criteria, comparability of cohorts and assessment of outcomes. By applying those criteria, we attributed poor quality (studies rating from 0 to 2 points), fair quality (studies rating from 3 to 5 points) and good/high quality (studies rating from 6 to 9 points) to each study.

### Clinical features

In addition to sex and age, we assessed the following key clinical features: 1) major cardiovascular risk factors (i.e., arterial hypertension, diabetes mellitus, dyslipidemia, cigarette smoking, coronary heart disease, peripheral arterial disease, history of prior stroke or transient ischemic attack [TIA], presence of AF), 2) stroke severity at admission (as measured using the National Institute of Health Stroke Scale (NIHSS) score, 3) acute stroke management (i.e., use of intravenous thrombolysis [IVT], endovascular treatment [EVT], and combined revascularization).

### Outcomes measures

The primary outcomes of interest were: 1) in-hospital and 2) 90-day mortality due to any cause; 3) 90-day functional independence, as defined by a modified Rankin Scale (mRS) score of 0 to 2; 4) 90-day ischemic stroke recurrence; 4) recurrent ischemic stroke at 90 days, as defined by a new ischemic cerebral event causing ischemic brain lesion(s) documented on imaging at 90 days post-index event.

5) intracranial hemorrhage (ICH), as defined by any intracranial bleeding or any category of hemorrhagic transformation at 24 to 48 h post-stroke brain neuroimaging, according to the Heidelberg classification system. [25]

### Data extraction

Data extraction was conducted by 2 authors, independently blinded (FG and FDS) and thereafter validated by a third author (MF), using an electronic spreadsheet with the following pre-specified variables: first author’s name, publication year, type of OAC (VKA or DOAC), total number of patients, proportion of males, mean age, proportion of patients with major cardiovascular risk factors, median NIHSS on admission, proportions of patients who underwent acute reperfusion therapies, proportion of patients with in-hospital and 90-day death due to any cause, ICH, 90-day functional independence (mRS of 0 to 2) and 90-day ischemic stroke recurrence. When an article reported separate data for ischemic stroke patients on DOAC or VKA, we included those patients in meta-analysis as distinct cohorts.

### Statistical analysis

To give a comprehensive account of the characteristics of ischemic strokes occurring during treatment with anticoagulants, we performed meta-analyses of general characteristics of included studies including age, sex distribution, vascular risk factors, severity (NIHSS score), and treatment (intravenous thrombolysis, endovascular treatment, or both) following a random-effects model [26]. We pooled binary data such as sex or presence of risk factors via meta-analyses of proportion, while we pooled continuous data via meta-analyses of means. Median values were converted to means according to standard formulas [27]. We also planned to assess the modifying effect of stroke severity, type of anticoagulant, and revascularization treatments on in-hospital death, mRS of 0–2 at 90 days, and ICH via meta-regression. Single parameters or entire meta-regressions were omitted in case of overfitting. To provide an account of the modifying effect of the chosen moderators, we reported the R [2] values for heterogeneity, corresponding to the estimated proportion of between-study heterogeneity that was attributable to the moderators. All analyses were stratified according to the type of OAC – DOACs or VKAs – if specified. We performed all analyses with R software, version 4.2.2, with the *meta* and *metafor* packages.

## Results

### Literature search

**Fig. 1:**
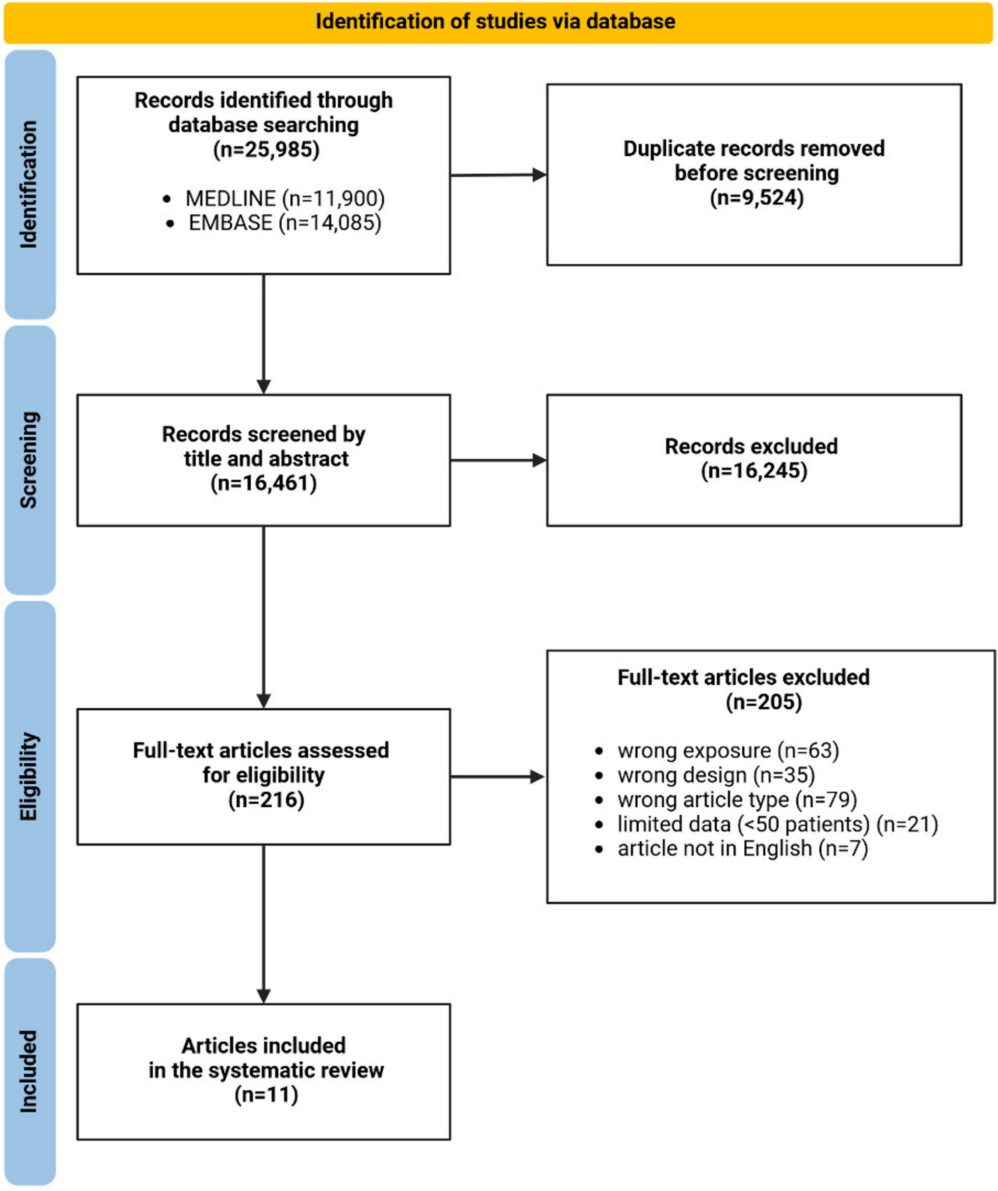
PRISMA flow-chart. Abbrev. DOAC: direct oral anticoagulant; INR: international normalized ratio; NA: not available; OAC: oral anticoagulation; VKA: vitamin K antagonist

Our search identified 25,985 articles. After removal of duplicates, 16,461 articles were screened for title and abstract; 216 were eligible for full-text screening. After full-text assessment, 11 articles reporting data on 14 cohorts were included in the meta-analysis. There was no overlap in the cohorts of the included studies. Figure 1 shows the PRISMA flow-chart of the literature search.

### Overview of the included study characteristics

The 11 included studies encompassed 10.624 patients with ischemic strokes on OACs (Supplementary Table 2). Three studies included two distinct patient subgroups, differentiated by treatment regimen (e.g., appropriate standard-dose vs. appropriate low-dose DOACs, or VKA in the therapeutic INR range vs. appropriate dose DOAC); Supplementary Table 2*.* Notably, among the VKA-treated patient studies included in our meta-analysis, one study categorized patients using INR values ≤ 1.7 versus > 1.7 [18], whereas the others defined therapeutic anticoagulation at the index stroke using an INR threshold of ≥ 2. Supplementary Table 2. Baseline clinicodemographic features and outcomes evaluated by individual studies are detailed in Supplementary Table 2 and 3, respectively. Notably, three studies did not report any outcome of interest or were deemed ineligible for outcomes meta-analysis due to inconsistent assessment or the application of different, non-comparable definitions [28–30]. However, these studies were included in the analysis to ensure a comprehensive assessment of the baseline characteristics of the study population.

### Baseline characteristics and outcomes of patients with ischemic stroke despite appropriate oral anticoagulation

Demographic and clinical characteristics of patients included in the meta-analysis are shown in Table 2 and in Supplementary Fig. 1. Overall, 55.2% (95% CI 47.8–62.4) of patients were males, while the mean age was 76.0 years (95% CI 73.2–78.7). All patients were on OAC for AF. However, among studies involving patients with ischemic stroke on VKAs, a single study reported that 586 patients (8.2%), out of a total of 7176 patients with AF, had coexisting mechanical heart valves [31]. Conversely, the remaining studies did not specify whether ischemic stroke patients treated with VKAs also had concomitant mechanical prosthetic valves. The median National Institute of Health Stroke Scale (NIHSS) score on admission was 5.1 (95% CI 5.0–5.3). The most prevalent vascular risk factors were arterial hypertension (78.3% [95% CI 72.2–83.3]), dyslipidemia (44.3% [95% CI 34.0–55.7]), and prior stroke or TIA (55.6% [95% CI 35.5–74.0]); Table 2. Outcomes are reported in Table 3 and in Supplementary Fig. 2. The pooled proportion of in-hospital deaths was 5.7% (95% CI 2.1–14.5). At 90 days, functional independence (mRS 0–2) was observed in 52.4% (95% CI 35.4–68.8) of patients, while mortality was 3.7% (95% CI 0.9–13.6)*.* A single study investigated the 90-day mortality rate among patients treated with VKAs within therapeutic INR level (≥ 2), reporting a rate of 3.7% [32]. The overall risk of ICH was 23.0% (95% CI 14.8–33.9). The 90-day risk of ischemic stroke recurrence was reported in 2 of the 11 included studies [28, 33]. However, neither study provided data specifically for patients who were appropriately anticoagulated at the time of their ischemic stroke. Benz et al. reported an overall 90-day stroke recurrence rate of 3.0% in a cohort of 1,163 patients; however, this population included warfarin users without reported INR values, making it impossible to isolate outcomes for appropriately anticoagulated individuals [28]. Similarly, O'Donnell et al. observed a 3% recurrence rate during the in-hospital stay, not over 90 days, among patients with ischemic stroke on warfarin who had a subtherapeutic INR (< 2). Data for patients with therapeutic INR levels (≥ 2) were not disclosed, reportedly to protect patient confidentiality due to the small sample size [33]. Additionally, a single study evaluated the risk of ICH in patients receiving DOACs reporting a 24-h ICH rate of 23%. [34]

**Table 2:** Pooled prevalence of demographic and clinical features overall and by type of oral anticoagulant

	Overall	DOACs	VKAs	p value for between-group differences
Males, % (95% CI)	55.2 (47.8–62.4)	57.4 (44.3–69.6)	53.2 (48.1–58.3)	0.559
Age, mean (95% CI)	76.0 (73.2–78.7)	76.0 (72.2–79.7)	76.1 (71.6–80.5)	0.959
Arterial hypertension, % (95% CI)	78.3 (72.2–83.3)	80.5 (73.7–85.9)	75.2 (64.3–83.6)	0.355
Diabetes mellitus, % (95% CI)	29.0 (24.1–34.3)	31.9 (25.2–39.6)	25.5 (19.6–32.4)	0.192
Cigarette smoking, % (95% CI)	17.6 (9.2–31.2)	23.5 (9.6–47.1)	11.6 (5.3–23.2)	0.216
Prior stroke or TIA, % (95% CI)	55.6 (35.5–74.0)	44.1 (41.0–47.5)	69.8 (28.3–93.2)	0.235
Dyslipidemia, % (95% CI)	44.3 (34.0–55.7)	40.5 (31.0–51.0)	47.2 (30.4–65.0)	0.522
Coronary heart disease, % (95% CI)	23.5 (13.7–37.2)	11.3 (5.2–23.0)	28.8 (18.3–42.1)	0.029
Peripheral arterial disease, % (95% CI)	31.0 (0.1–75.8)	37.9 (2.8–92.8)	23.1(4.8–64.4)	0.696
Atrial fibrillation, % (95% CI)	100.0 (98.6–100.0)	100.0 (99.3–100.0)	100.0 (0.2–100.0)	0.999
NIHSS score, mean (95% CI)	5.1 (5.0–5.3)	5.7 (2.8–8.5)	5.1 (5.0–5.3)	0.713
Intravenous thrombolysis, % (95% CI)	0.3 (0.1–2.0)	0.3 (0.0–7.2)	0.3 (0.0–2.1)	0.962
Endovascular treatment, % (95% CI)	0.5 (0.0–16.4)	0.5 (0.0–2.5)	0.5 (0.1–2.5)	0.980
Combined revascularization, % (95% CI)	0.0 (0.0–9.7)	0.3 (0.0–9.3)	0.0 (0.0–100.0)	0.999

CI: confidence interval; NIHSS: National Institutes of Health Stroke Scale; TIA: transient ischemic attack

All tests are performed according to a random-effect meta-analytical model

**Table 3:** Outcomes of patients with ischemic stroke on treatment with oral anticoagulants

	Overall	DOACs	VKAs	p value for between-group differences
In-hospital death	5.7 (2.1–14.5)	2.7 (0.7–10.2)	7.1 (2.4–19.0)	0.272
Death at 90 days	3.7 (0.9–13.6)	No data	3.7 (0.9–13.6)	-
90-day mRS 0–2	52.4 (35.4–68.8)	60.4 (36.0–80.5)	35.7 (32.7–38.8)	0.048
ICH	23.0 (14.8–33.9)	23.0 (14.8–33.9)	No data	-

Abbrev. DOACs: direct oral anticoagulants; ICH: intracranial hemorrhage; mRS: modified Rankin Scale; VKAs: vitamin k antagonists

Data are provided as proportions with 95% confidence intervals (CIs)

### Comparison of baseline characteristics and outcomes between patients on DOAC versus VKA

**Fig. 2:**
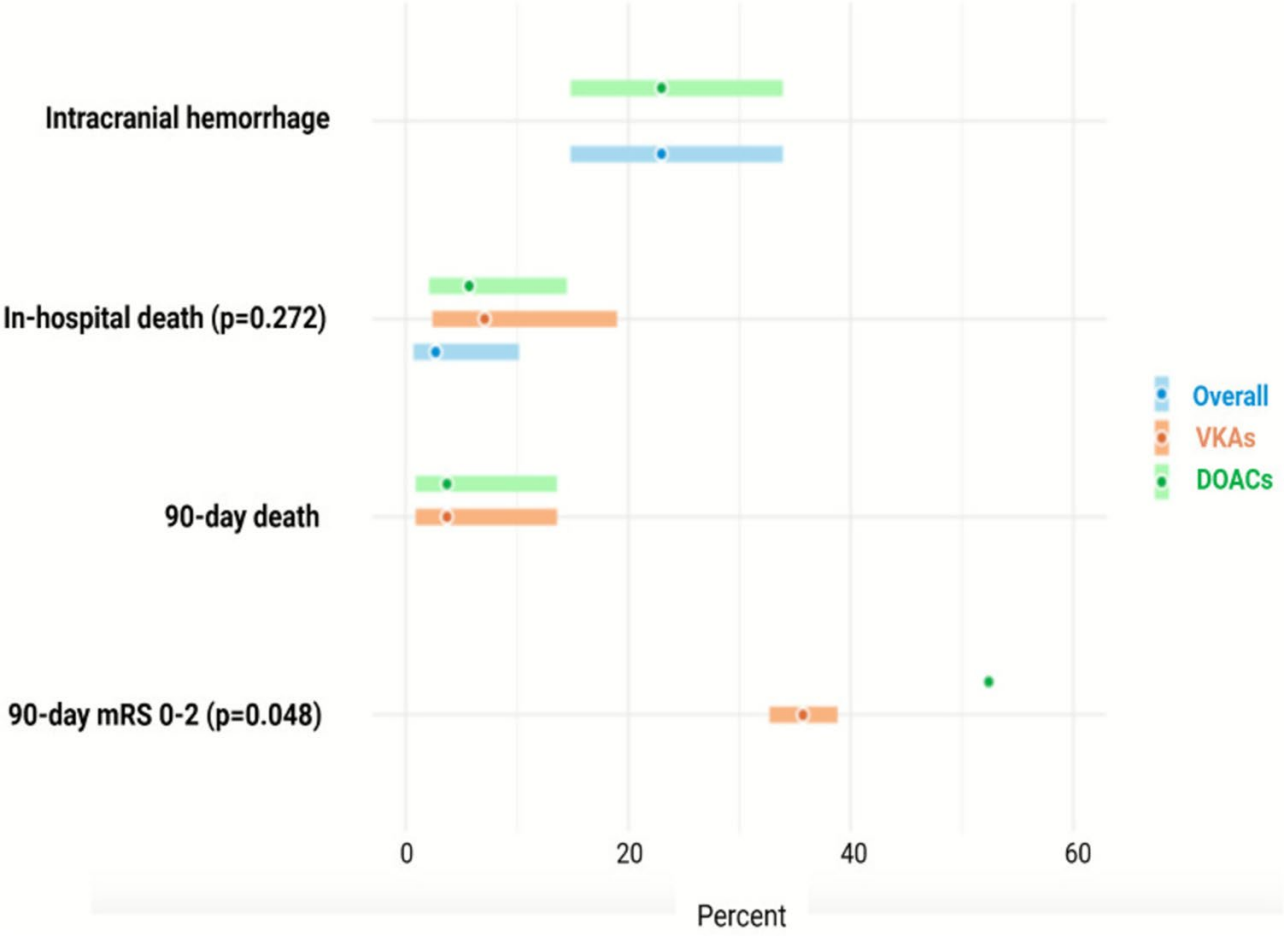
Outcomes of patients with ischemic stroke on treatment with oral anticoagulants. Abbrev. mRS: modified Rankin Scale; DOACs: direct oral anticoagulants; VKAs: vitamin K antagonists. Data are provided as proportions with 95% confidence intervals (CIs). *p-value not available (no data for VKAs)

Regarding the baseline characteristics, there were no significant differences according to OAC type, except for coronary heart disease, which was more prevalent in the VKA group (28.8% [95% CI 18.3–42.1]) compared to the DOAC group (23.5% [95% CI 13.7–37.2]; p = 0.029). In terms of outcomes, ischemic strokes occurring on DOAC showed a significantly higher proportion of favorable outcomes (mRS 0–2 at 90-days) compared with those treated with VKAs (p = 0.048); Table 3, Fig. 2.

### Meta-regression

Meta-regression results for in-hospital death and 90-day mRS score of 0 to 2 are shown in Table 4. There was no significant effect of sex, age or anticoagulant type on these outcomes. Meta-regression analyses for 90-day death and ICH were omitted as not estimable.

**Table 4:** Moderators for effect on in-hospital death and modified Rankin Scale (mRS) score of 0–2 at 90 days

In-hospital death	Estimate	Standard error	Z-value	p value	Confidence interval
Intercept	−0.6001	0.3373	−1.7791	0.075	−1.2611 to 0.0610
Sex (males)	1.4062	0.7484	1.8789	0.060	−0.0607 to 2.8730
Type of anticoagulant					
DOACs (ref)	-	-	-	-	-
VKAs	0.0203	0.0601	0.3377	0.736	−0.0976 to 0.1382
90-day mRS 0–2	Estimate	Standard error	Z-value	p value	Confidence interval
Intercept	1.6803	1.2099	1.3888	0.165	−0.6910 to 4.0517
Sex (males)	0.3199	0.5049	0.6337	0.526	−0.6696 to 1.3095
Age	−0.0171	0.0123	−1.3905	0.164	−0.0411 to 0.0070
Type of anticoagulant					
DOACs (ref)	-	-	-	-	-
VKAs	−0.1622	0.1199	−1.3531	0.176	−0.3971 to 0.0727

Abbrevi. DOACs: direct oral anticoagulants; mRS: modified Rankin Scale; VKAs: vitamin k antagonists

Data are provided as proportions with 95% confidence intervals (CIs)

### Quality assessment

Among the 11 studies included in this meta-analysis, all were classified as high quality (scoring between 6 and 9 points). For single-cohort studies without a control group, the "Selection of the Non-Exposed Cohort" criterion from the Newcastle–Ottawa Scale was considered non-assessable. This adaptation ensured an equitable assessment of methodological quality across different study designs; Supplementary Fig. 3.

## Discussion

Our systematic review and meta-analysis provided a comprehensive assessment of the clinicodemographic profile and outcomes of ischemic stroke in patients receiving therapeutic OACs at appropriate doses, with a particular focus on comparing ischemic stroke cases occurring with DOACs versus VKAs.

A key priority in managing patients who experience an ischemic stroke while on OACs is to determine whether they present with unique demographic or clinical characteristics that can aid in identifying underlying risk factors and optimizing therapeutic strategies. To this end, we pooled not only outcomes, but also baseline characteristics across the available real-world studies. The pooled analysis of the available data showed a higher mean age (76 years) and a greater prevalence of previous ischemic stroke (55.6%) compared with the general population of patients with ischemic stroke [35, 36]. This finding is not surprising, as oral anticoagulants are usually prescribed after a first ischemic stroke attributable to AF for secondary prevention, or following the detection of AF, which is common in the elderly [37–39]. Furthermore, our cohort, representing a more real-world context, significantly differs from those typically included in major anticoagulant trials, which often involve younger populations (average age ranging from 70 to 73 years), a lower male proportion (approximately 52%), and fewer comorbidities (hypertension 60–65% and dyslipidemia 35–40%) [40–43]. Importantly, our study included patients with recurrent ischemic strokes, whereas most trials focused on individuals without a history of prior stroke.

The comparison between patients treated with DOACs and those treated with VKAs revealed higher stroke severity in the former compared to the latter group. This finding contrasts with observational studies, which did not find differences in stroke severity between patients treated with DOACs and those treated with VKAs [44, 45]. Populations treated with VKAs and DOACs were included in different periods, as DOACs were introduced for clinical use more recently than VKAs. Additionally, prescribing patterns might have varied between DOACs and VKAs, leading to potential selection bias.

Referring to outcomes, we found an in-hospital death rate of 5.7%, which was 3.7% at 90 days. The discrepancy in case-fatality—higher at a shorter time point and lower at a longer time point—is due to the different numbers of studies that were included in meta-analyses. The most robust data are those regarding in-hospital death as more studies reported those data compared death at 90 days. Specifically, among the included studies, in-hospital mortality was reported for a total of 7,491 patients, whereas 90-day mortality was available for only 54 patients, as reported by a single study [46]. This inconsistency underscores a significant gap in the literature regarding both short- and long-term outcomes in this population and highlights the urgent need for further research to address this limitation. The in-hospital mortality rates of patients with ischemic stroke while on oral anticoagulants were comparable to the 5% rates found in the general population of patients with ischemic stroke [47–49]. Patients with ischemic stroke while on DOACs had a higher proportion of functional independence (mRS score of 0 to 2) compared to those with ischemic stroke while on VKAs (60.4% vs 35.7%). Conversely, we found comparable case-fatality rates between DOACs and VKAs. Our findings regarding 90-day functional outcomes are consistent with previous studies that reported more favorable outcomes associated with DOAC use compared to VKAs [50, 51]. In contrast, our mortality data diverge from the ARISTOTLE trial, which showed a lower mortality rate with apixaban (3.52% per year) compared to warfarin (3.94% per year) [42]. This discrepancy may be attributed to differences in patient selection between the populations treated with DOACs and VKAs in the studies included in our analysis, which focused exclusively on ischemic stroke patients receiving therapeutic doses of OACs.

Our meta-regression analyses suggest no significant difference between DOACs and VKAs in determining patients’ outcomes. Nevertheless, we should keep in mind that our included studies collected data from clinical practice in which patients were selected by clinicians and not randomized. It should be noted that our data were very heterogeneous, which may have impacted on meta-regression analyses. As shown by the low R^2^ values, the moderators of outcomes accounted for a very limited proportion of the observed heterogeneity, whereas most of it was likely due to the heterogeneous data sources and collection. Therefore, while our descriptive data hold significant value as pooled analyses of available real-world evidence, comparisons between DOACs and VKAs should be interpreted with caution.

Our study has several limitations that should be considered when interpreting our findings. First, we could not account for the substantial variability among the included populations. In particular, differences in INR thresholds across studies involving AF patients on VKAs represent a key limitation. Furthermore, although all patients had AF, we were unable to distinguish between those with valvular and non-valvular AF in most studies. This is clinically relevant, as VKAs remain the standard of care for valvular AF, whereas DOACs are recommended for non-valvular AF. Only one VKA-based study reported the proportion of patients with mechanical heart valves. Therefore, we cannot exclude the possibility that some VKA-treated patients had valvular AF, potentially influencing clinical outcomes. Another limitation concerns the absence of plasma OAC concentration measurements. None of the included studies reported such data, reflecting the limited use of drug-level monitoring in current clinical practice. All studies relied on guideline-recommended, on-label dosing, without biochemical confirmation of anticoagulant activity. This highlights a broader gap in the literature and underscores the need to standardize analytical methods for DOAC level assessment to better understand the relationship between plasma concentration and clinical outcomes. Moreover, we could not evaluate long-term outcomes following ischemic stroke on OACs or account for adherence and compliance—factors known to differ between DOAC and VKA users and to affect real-world efficacy. We also could not analyze potential differences between DOAC subtypes or among specific VKAs, which may have important clinical implications. Importantly, none of the included studies provided data on recurrent ischemic stroke at 90 days following the index event, limiting our ability to assess the risk of recurrence in this population. Similarly, none of the studies assessed anticoagulant activity at the time of stroke onset, which may have resulted in misclassification of some cases as appropriately anticoagulated despite possible subtherapeutic levels. Finally, stroke etiology was not systematically reported, and it is plausible that a proportion of strokes occurring under appropriate anticoagulation were due to competing mechanisms rather than cardioembolism from AF. This uncertainty further limits the interpretability of outcome data and highlights the need for future research that includes detailed stroke etiological classification.

In conclusion, our systematic review and meta-analysis provide a comprehensive synthesis of real-world observational data on ischemic stroke patients treated with OAC at guideline-recommended doses and for appropriate indications. By focusing specifically on this underrepresented cohort, our study offers a novel perspective distinct from previous analyses that often include patients on off-label or subtherapeutic OAC regimens. This approach allows for a more accurate assessment of ischemic risk despite appropriate anticoagulation. Our findings indicate that ischemic stroke in this population is associated with a high burden of vascular risk factors and moderate stroke severity. Notably, patients receiving DOACs demonstrated more favorable 90-day functional outcomes compared to those on VKAs. Importantly, our analysis highlights substantial gaps in the current literature, particularly regarding long-term outcomes and the underlying mechanisms of stroke despite adequate anticoagulation. These gaps underscore the need for larger, well-designed prospective studies to identify predictors of poor prognosis, clarify stroke etiologies in anticoagulated patients, and ultimately guide the development of more effective secondary prevention strategies.

## Supplementary Information

Below is the link to the electronic supplementary material.

Supplementary file1 (PDF 858 KB)

Supplementary file2 (DOCX 22 KB)

## Data Availability

The dataset used for this meta-analysis will be shared upon request from any qualified researcher to the corresponding author.
