# Supplementary material for: Clinical characteristics and outcomes of ischemic stroke despite appropriate oral anticoagulation for atrial fibrillation: A systematic review and meta-analysis of real-world studies
Source: Neurol Sci. 2025 Dec 22;47(1):27. doi: 10.1007/s10072-025-08734-2 (PMC12722486; doi:10.1007/s10072-025-08734-2)
Supplement: Supplementary file 1 — Supplementary file1 (PDF 858 KB) [file 10072_2025_8734_MOESM1_ESM.pdf]

Supplemental Figure 1. Forest plots of baseline characteristics in the included studies.

1.1 Sex (males)

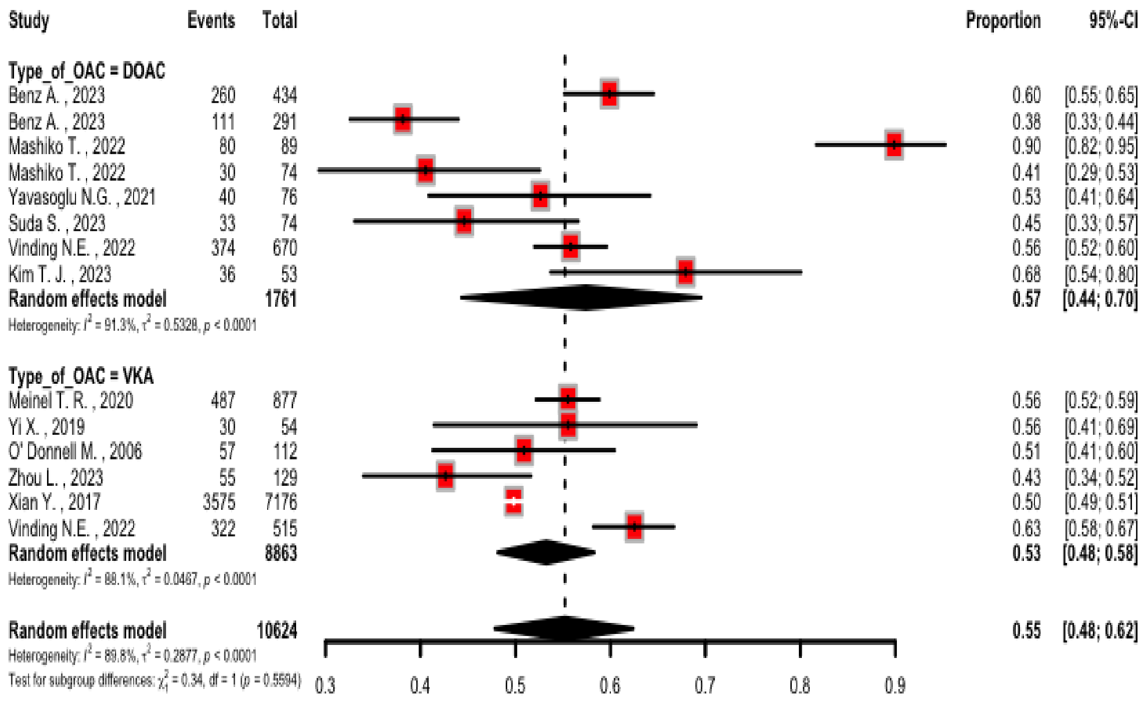

1.2 Age

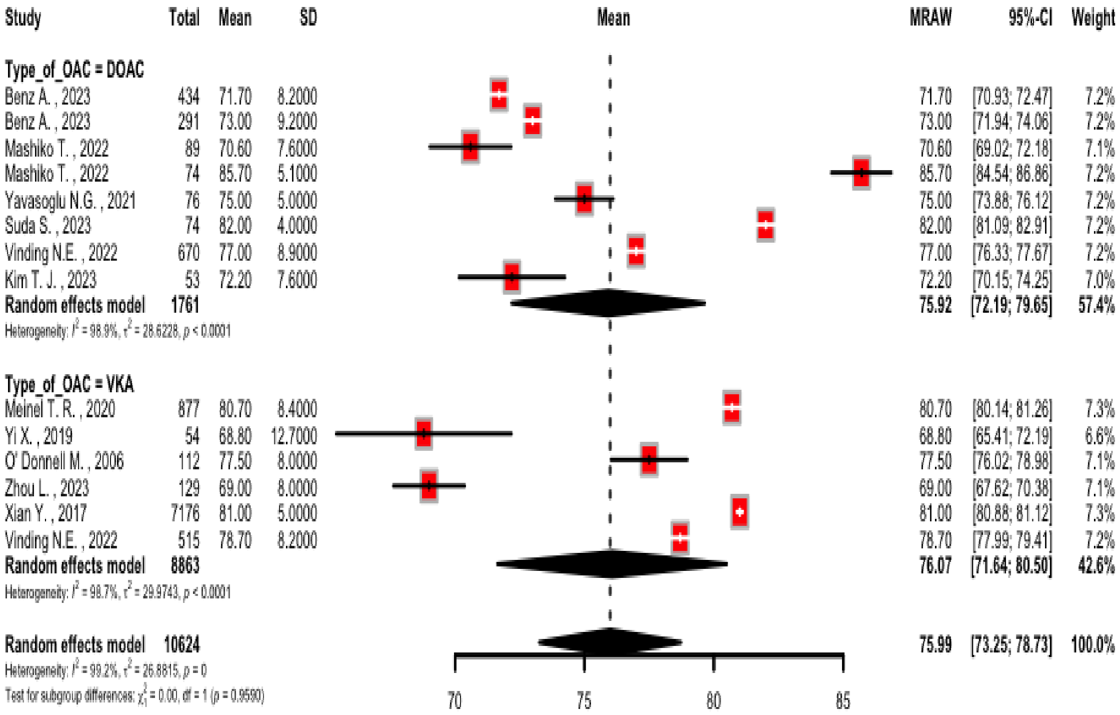

Study Total Mean SD
Mean
MRAW 95%-CI Weight
Type_of_OAC = DOAC
Benz A. , 2023 434 71.70 8.2000 71.70 [70.93; 72.47] 7.2%
Benz A. , 2023 291 73.00 9.2000 73.00 [71.94; 74.06] 7.2%
Mashiko T. , 2022 89 70.60 7.6000 70.60 [69.02; 72.18] 7.1%
Mashiko T. , 2022 74 85.70 5.1000 85.70 [84.54; 86.86] 7.2%
Yavasoglu N.G. , 2021 76 75.00 5.0000 75.00 [73.88; 76.12] 7.2%
Suda S. , 2023 74 82.00 4.0000 82.00 [81.09; 82.91] 7.2%
Vinding N.E. , 2022 670 77.00 8.9000 77.00 [76.33; 77.67] 7.2%
Kim T. J. , 2023 53 72.20 7.6000 72.20 [70.15; 74.25] 7.0%
Random effects model 1761 75.92 [72.19; 79.65] 57.4%
Heterogeneity: I^2 = 98.9%, τ^2 = 28.6228, p < 0.0001
Type_of_OAC = VKA
Meinel T. R. , 2020 877 80.70 8.4000 80.70 [80.14; 81.26] 7.3%
Yi X. , 2019 54 68.80 12.7000 68.80 [65.41; 72.19] 6.6%
O' Donnell M. , 2006 112 77.50 8.0000 77.50 [76.02; 78.98] 7.1%
Zhou L. , 2023 129 69.00 8.0000 69.00 [67.62; 70.38] 7.1%
Xian Y. , 2017 7176 81.00 5.0000 81.00 [80.88; 81.12] 7.3%
Vinding N.E. , 2022 515 78.70 8.2000 78.70 [77.99; 79.41] 7.2%
Random effects model 8863 76.07 [71.64; 80.50] 42.6%
Heterogeneity: I^2 = 98.7%, τ^2 = 29.9743, p < 0.0001
Random effects model 10624 75.99 [73.25; 78.73] 100.0%
Heterogeneity: I^2 = 99.2%, τ^2 = 26.8815, p = 0
Test for subgroup differences: χ^2_1 = 0.00, df = 1 (p = 0.9590)
70 75 80 85

1.3 Arterial hypertension

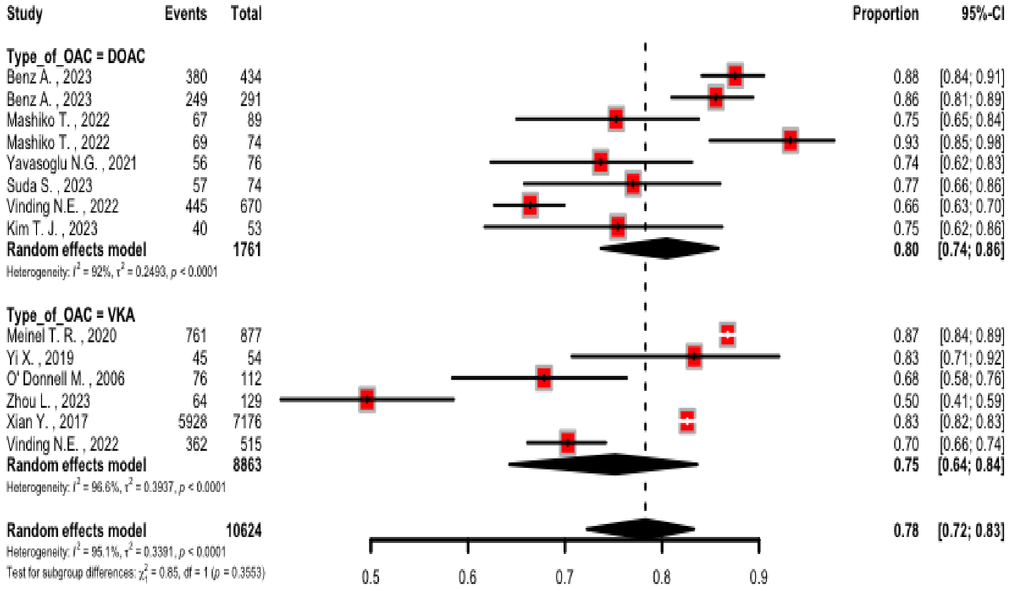

1.4 Diabetes mellitus

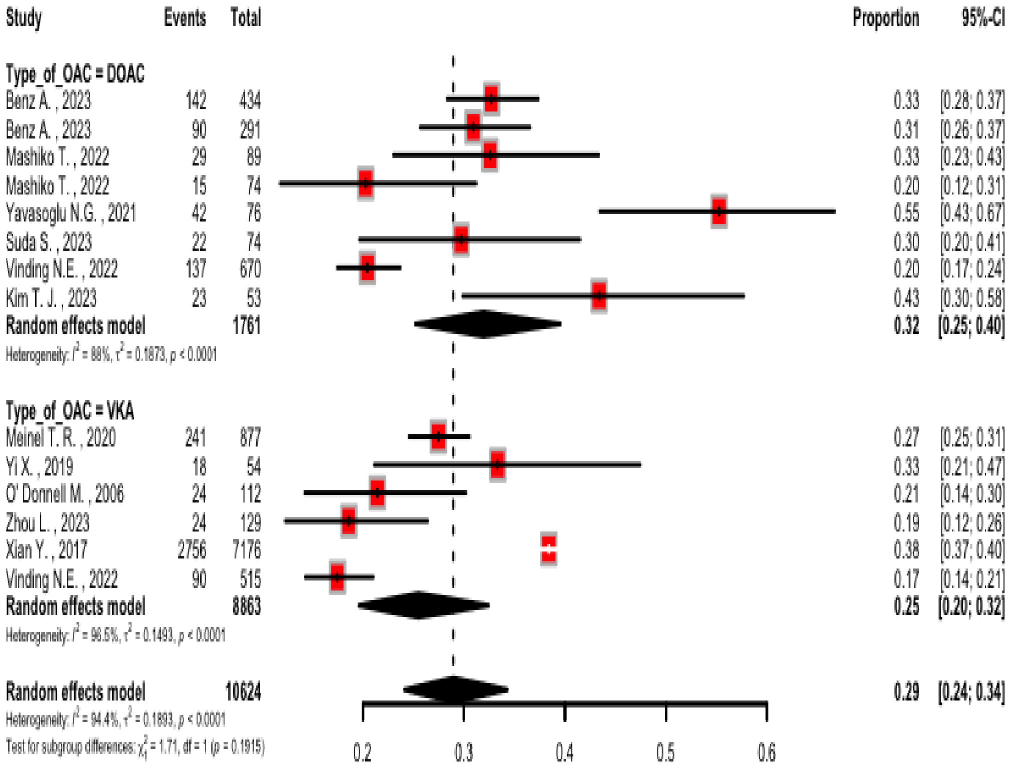

1.5 Cigarette smoking

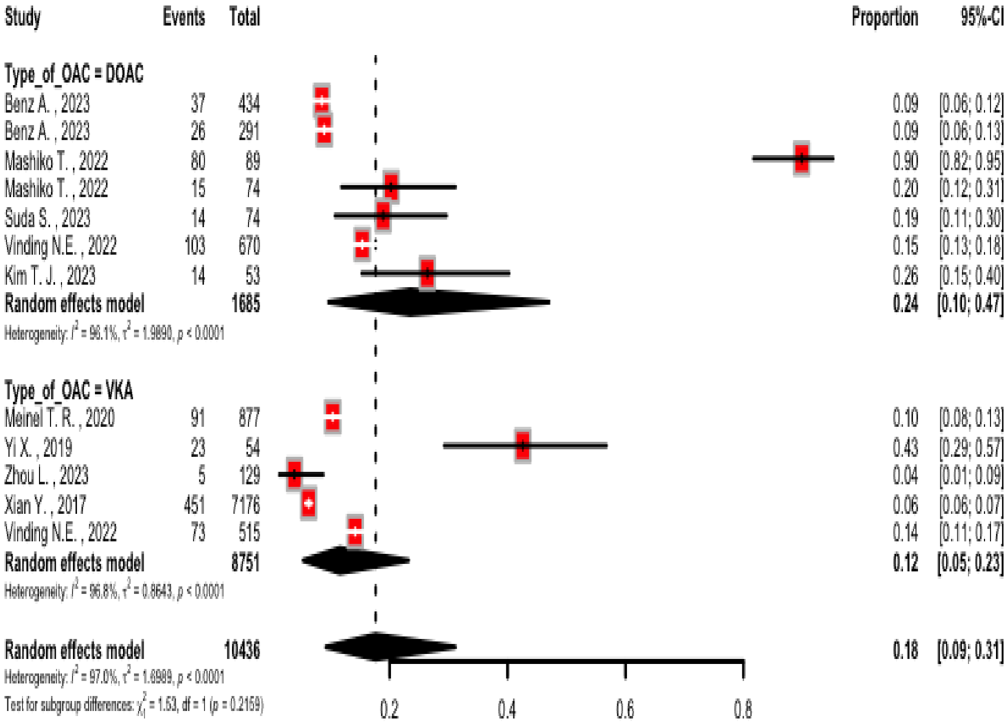

1.6 Prior stroke or TIA

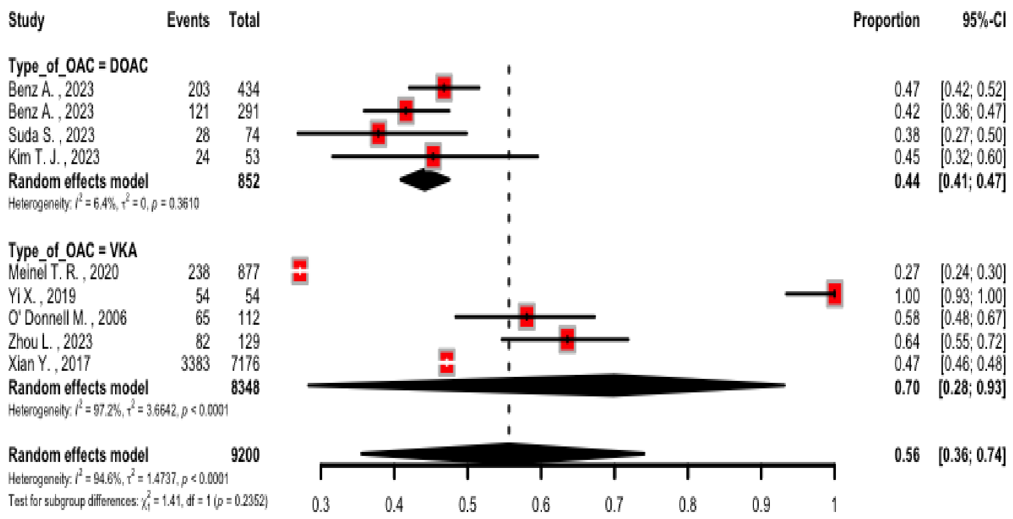

1.7 Dyslipidemia

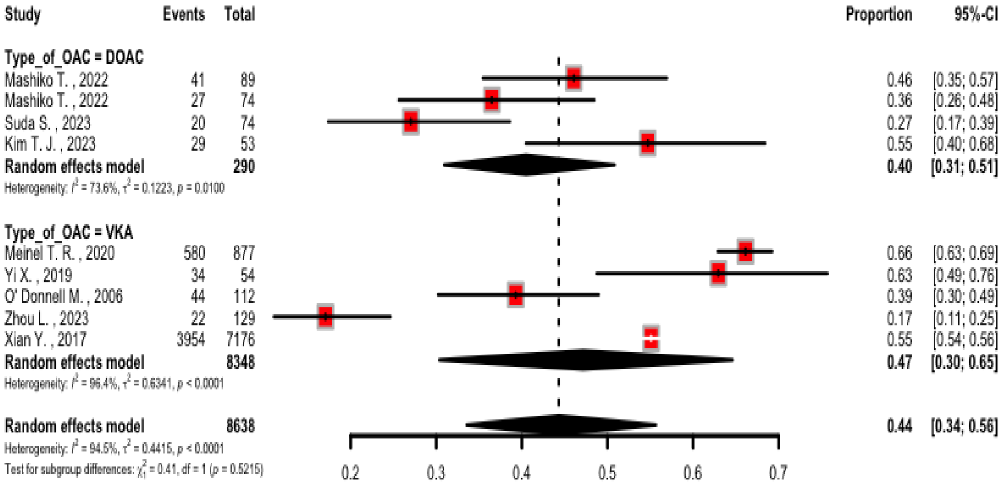

1.8 Coronary heart disease

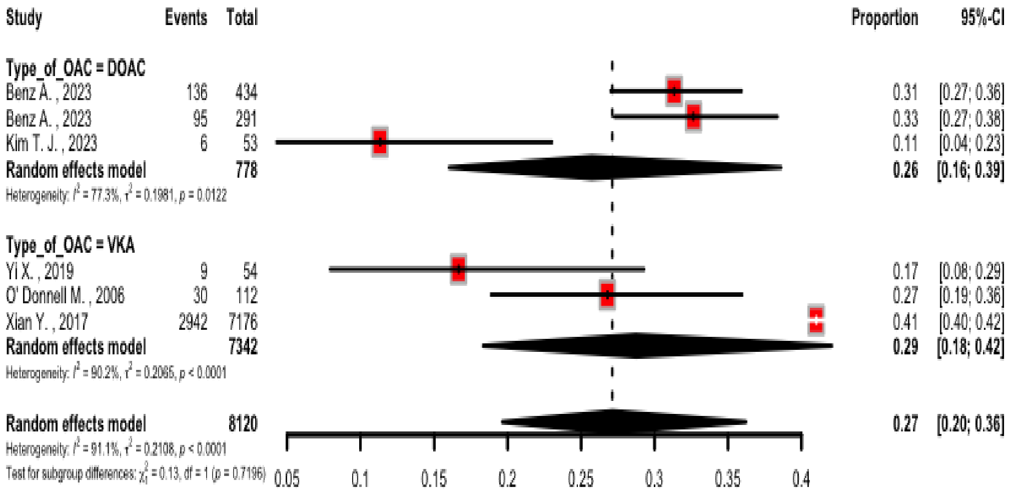

1.9 Peripheral arterial disease

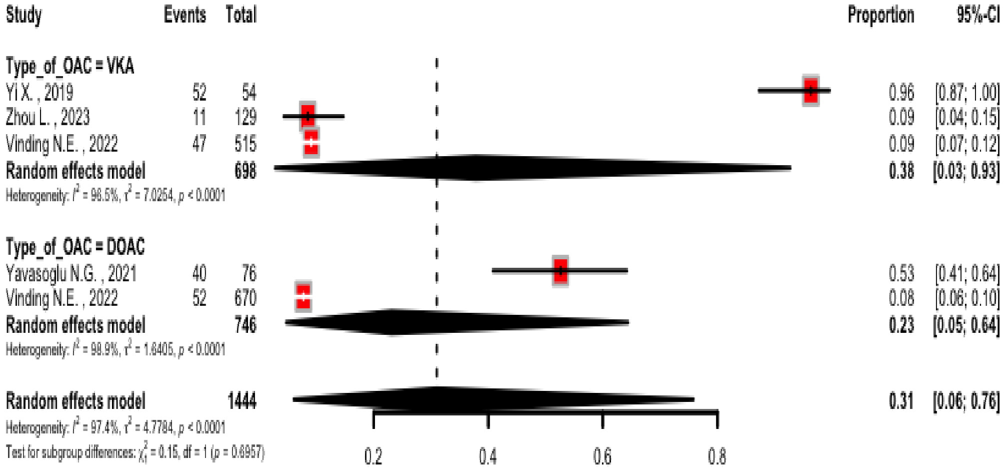

1.10 Atrial fibrillation

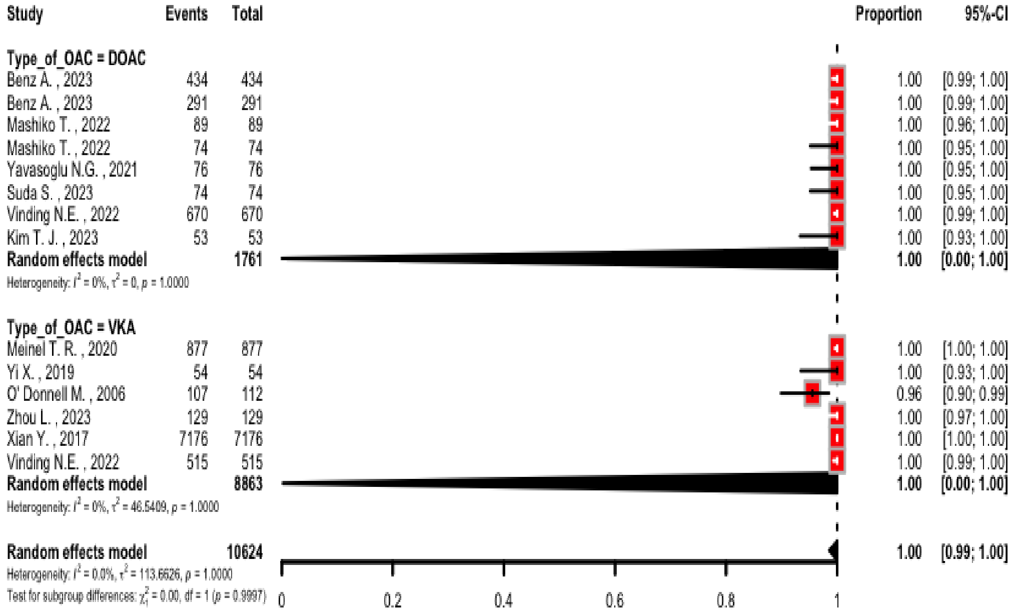

1.11 Stroke severity (National Institute of Health Stroke Scale [NIHSS] score)

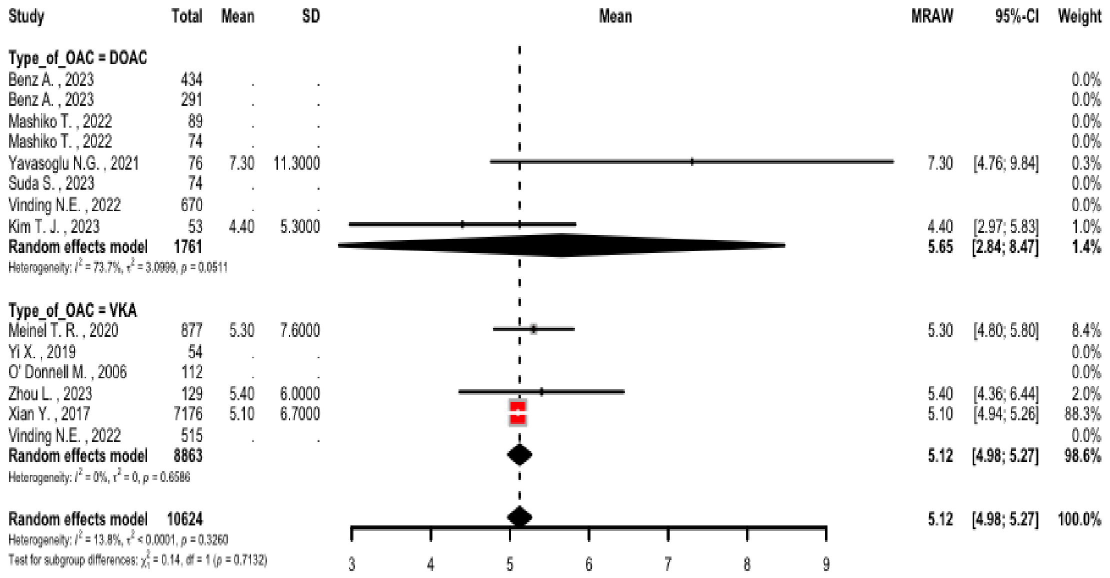

1.12 Intravenous thrombolysis

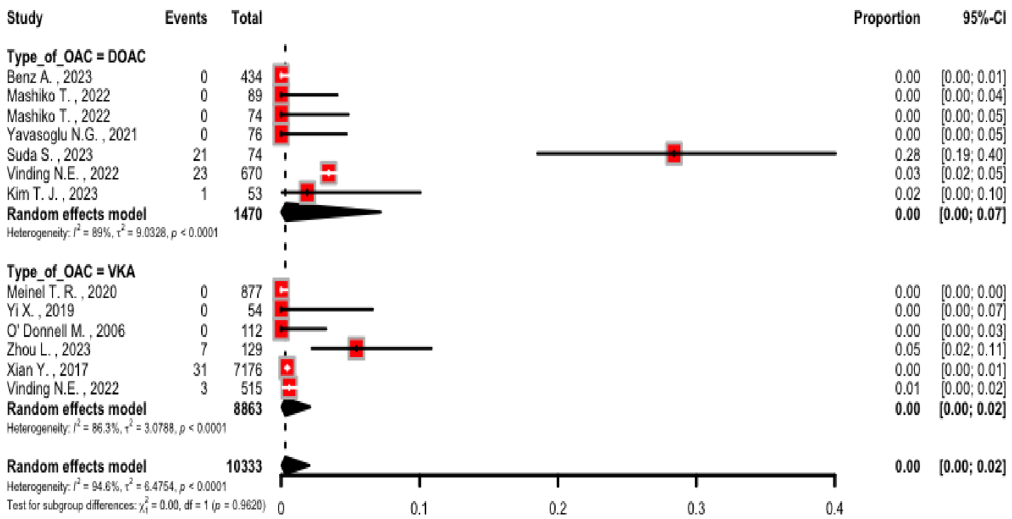

1.13 Endovascular thrombectomy

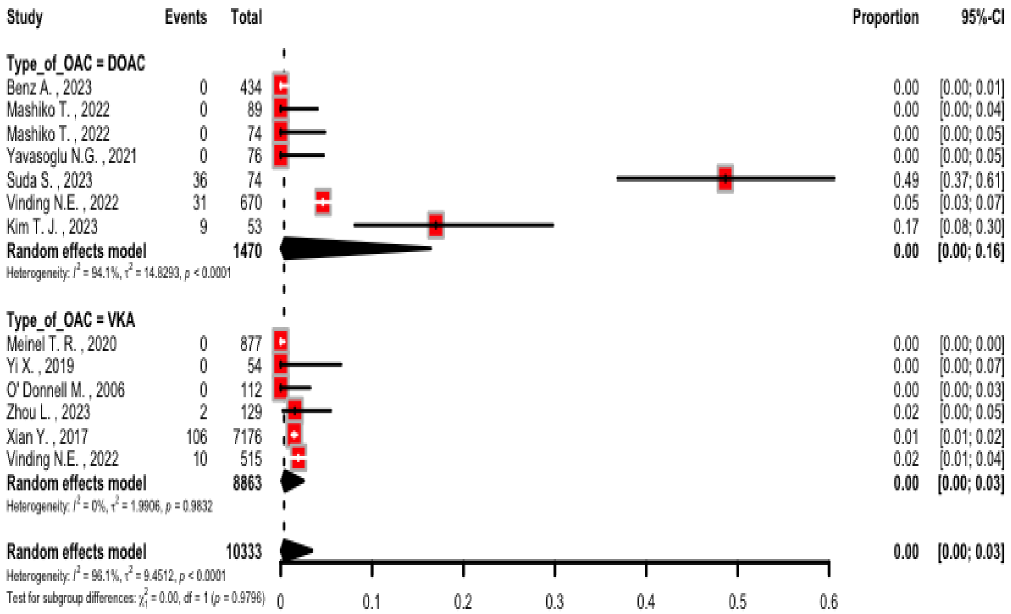

1.14 Combined (intravenous thrombolysis + endovascular thrombectomy)

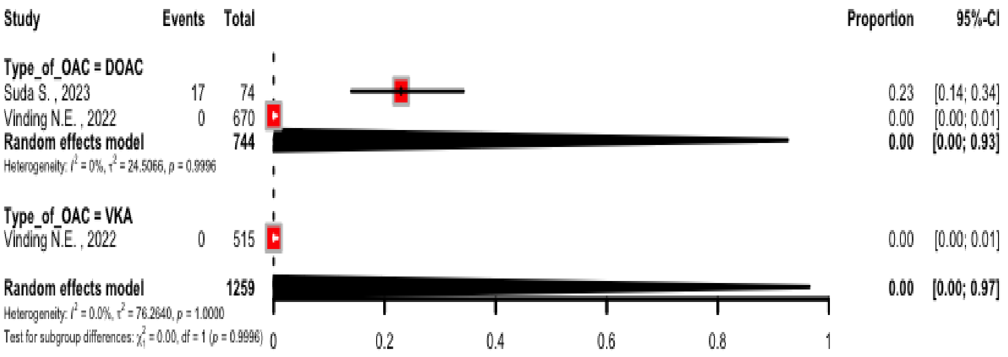

Supplementary Figure 2. Outcomes of patients with ischemic stroke on oral anticoagulants.

2.1 In-hospital death

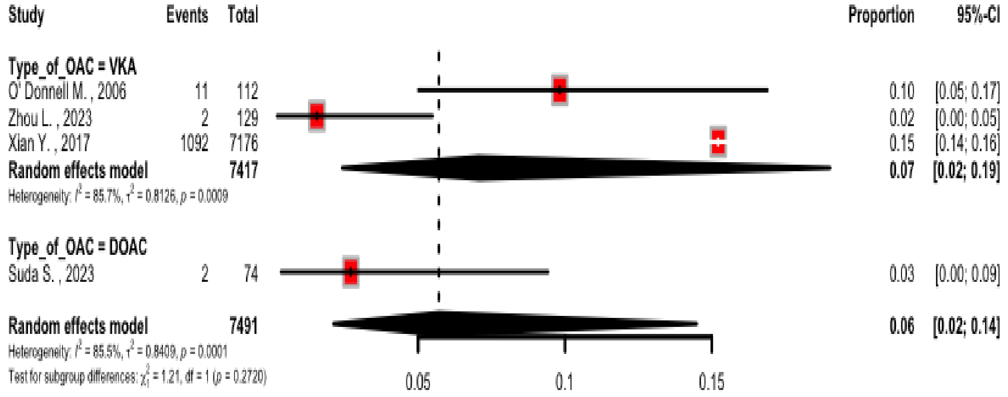

2.2 90-day death

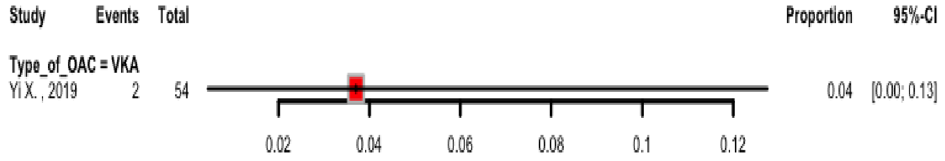

2.3 90-day mRS score of 0 to 2

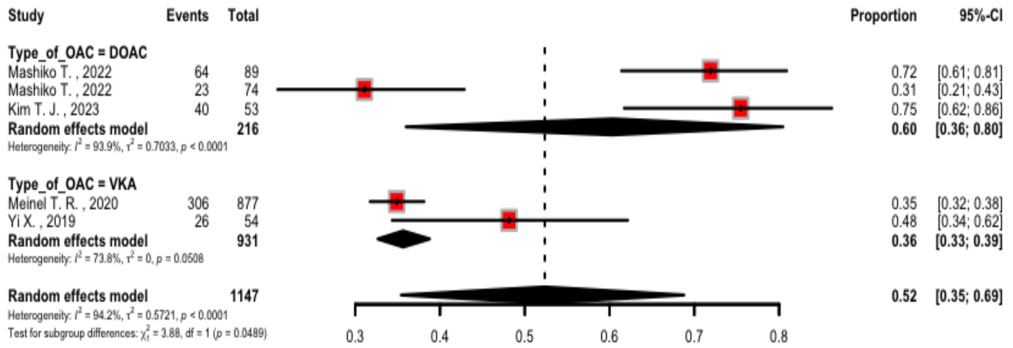

2.4 Intracranial hemorrhage

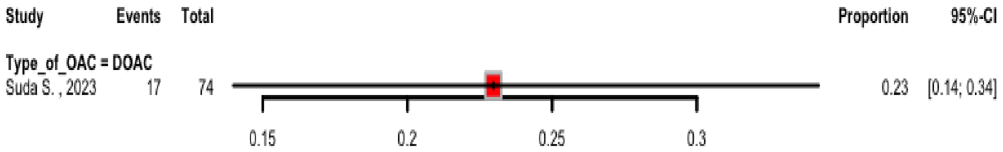

Supplemental Figure 3. Risk of bias

Author, Year	Selection (Max 4 stars)				Comparability (Max 2 stars)	Outcome/exposure (Max 3 stars)			Overall
	**Representativeness of the exposed cohort**	**Selection of the non exposed cohort**	**Ascertainment of exposure**	**Outcome not present at the start**	**Control of important and additional factors**	**Assessment of outcome**	**Adequacy of follow-up length**	**Adequacy of follow-up of cohorts**	
Benz et al., 2023	★	★	★	★	★	★	★		7/9
Kim TJ et al., 2023	★	★	★	★	★★	★	★	★	9/9
Mashiko et al., 2022	★	-	★	★	★★	★	★	★	8/9
Thomas Raphael Meinel et al., 2020	★	★	★	★	★★	★	★	★	9/9
Martin O' Donnell et al., 2006	★	★	★	★	★	★	★	★	8/9
Suda et al, 2023	★	-	★	★	★★	★	★	★	8/9
Vinding et al, 2022	★	-	★	★	★★	★	★	★	8/9
Xian et al, 2017	★	★	★	★	★★	★	★	★	9/9
Yavasoglu et al, 2021	★	★	★	★	★★	★	★	★	9/9
Yi et al, 2019	★	★	★	★	★	★	★	★	8/9
Zhou et al, 2023	★	★	★	★	★★	★	★	★	9/9
