# Supplementary material for: Clinical characteristics and outcomes of ischemic stroke despite appropriate oral anticoagulation for atrial fibrillation: A systematic review and meta-analysis of real-world studies
Source: Neurol Sci. 2025 Dec 22;47(1):27. doi: 10.1007/s10072-025-08734-2 (PMC12722486; doi:10.1007/s10072-025-08734-2)
Supplement: Supplementary file 2 — Supplementary file2 (DOCX 22 KB) [file 10072_2025_8734_MOESM2_ESM.docx]

# **Supplementary Table 1. Search strings.**

| **PubMed (MEDLINE)** |
| --- |
| ((ischemic stroke [Title/Abstract]) OR (acute ischemic stroke [Title/Abstract])) OR (stroke[Title/Abstract]) AND ((oral anticoagulants [Title/Abstract]) OR (oral anticoagulation [Title/Abstract]) OR (vitamin k antagonist [Title/Abstract]) OR (direct oral anticoagulants [Title/Abstract]) OR (warfarin [Title/Abstract]) OR (new oral anticoagulants [Title/Abstract]) OR (dabigatran [Title/Abstract]) OR (apixaban [Title/Abstract]) OR (edoxaban [Title/Abstract]) OR (rivaroxaban [Title/Abstract]))). |
| **Scopus** |
| TITLE-ABS (((ischemic AND stroke) OR (acute AND stroke) OR (acute AND ischemic AND stroke)) AND ((oral AND anticoagulants) OR (oral AND anticoagulation) OR (vitamin AND k AND antagonist) OR (direct AND oral AND anticoagulants) OR (warfarin) OR (new AND oral AND anticoagulants) OR (dabigatran) OR (apixaban) OR (edoxaban) OR (rivaroxaban))) |

**Supplementary Table 2***.* **Baseline characteristics of individual studies included in the meta-analysis.**

|  | **N** | **OAC type** | **AH** | **DM** | **CS** | **Prior stroke or TIA** | **Dyslipidemia** | **CHD** | **PAD** | **AF** | **Admission NIHSS** | **IVT** | **EVT** | **IVT + EVT** |
| --- | --- | --- | --- | --- | --- | --- | --- | --- | --- | --- | --- | --- | --- | --- |
| Benz A., 2023 | 434 | DOAC^a^ | X | X | X | X |  | X |  | X |  |  |  |  |
| Benz A., 2023 | 291 | DOAC^a^ | X | X | X | X |  | X |  | X |  |  |  |  |
| Kim T. J.,  2023 | 53 | DOAC | X | X | X | X | X | X |  | X | X | X | X |  |
| Mashiko T., 2022 | 89 | DOAC^b^ | X | X | X |  |  |  |  | X |  |  |  |  |
| Mashiko T., 2022 | 74 | DOAC^b^ | X | X | X |  |  |  |  | X |  |  |  |  |
| Meinel T. R.,2020 | 877 | VKA^c^ | X | X | X | X | X |  |  | X | X |  |  |  |
| O’ Donnel M., 2006 | 112 | VKA^d^ | X | X |  | X | X | X |  | X |  |  |  |  |
| Suda S., 2023 | 74 | DOAC^e^ | X | X | X | X | X |  |  | X | X | X | X | X |
| Vinding N.E., 2022 | 515 | VKA^f^ | X | X | X |  |  |  | X | X |  | X | X |  |
| Vinding N.E., 2022 | 670 | DOAC^g^ | X | X | X |  |  |  | X | X |  | X | X |  |
| Xian Y., 2017 | 7176^*^ | VKA^h^ | X | X | X | X | X | X |  | X | X | X | X |  |
| Yavasoglu N.G., 2021 | 76 | DOAC | X | X |  |  |  |  | X | X | X |  |  |  |
| Yi X., 2019 | 54 | VKA^i^ | X | X | X | X | X | X | X | X |  |  |  |  |
| Zhou L., 2023 | 129 | VKA^l^ | X | X | X | X | X |  | X | X | X | X | X |  |

Abbreviations. AH: arterial hypertension; AF: atrial fibrillation; CHD: coronary heart disease; CS: cigarette smoking; DM: diabetes mellitus; EVT: endovascular treatment; IVT: intravenous thrombolysis; N: number of patients from each study included in this meta-analysis; TIA: transient ischemic attack; PAD: peripheral arterial disease.

a. appropriate DOAC standard-dose (N=434); appropriate DOAC low-dose (N=291). b. appropriate DOAC standard-dose (N=89); appropriate DOAC low-dose (N=74). c. INR >1.7. d. INR ≥2. e. appropriate DOAC dose. f. INR 2-3. g. appropriate DOAC dose. h. INR ≥2. i. INR ≥2. l. INR ≥2.

^*^586 patients (8.2%), out of a total of 7176 patients with atrial fibrillation on VKA, had coexisting mechanical heart valves.

**Supplementary Table 3. Outcomes measured in individual studies included in the meta-analysis.**

|  | **In-hospital death** | **mRS 0-2  at 90 days** | **Death  at 90 days** | **ICH** | **90-day ischemic stroke recurrence** |
| --- | --- | --- | --- | --- | --- |
| Benz A., 2023 |  |  |  |  | X^a^ |
| Kim T.J., 2023 |  | X |  |  |  |
| Mashiko T., 2022 |  | X |  |  |  |
| Meinel T. R.,2020 |  | X |  |  |  |
| O’ Donnell M., 2006 | X |  |  |  | X^a,b^ |
| Suda S., 2023 | X |  |  | X |  |
| Vinding N.E., 2022 |  |  |  |  |  |
| Xian Y., 2017 | X |  |  |  |  |
| Yavasoglu N.G., 2021 |  |  |  |  |  |
| Yi X., 2019 |  | X | X |  |  |
| Zhou L., 2023 | X |  |  |  |  |

Abbreviations. mRS: modified Rankin scale; ICH: intracranial hemorrhage.

a. this outcome was not assessed in appropriately anticoagulated patients. b. this study evaluated ischemic stroke recurrence during in-hospital stay.
